# Decreased Expression of PLD2 Promotes EMT in Colorectal Cancer Invasion and Metastasis

**DOI:** 10.7150/jca.89970

**Published:** 2024-03-31

**Authors:** Xuan Liu, Lei Shen, Haiyu Wang

**Affiliations:** 1 Department of General Surgery, Central Hospital of Xuhui District, Shanghai, China.; 2 Department of General Surgery, Zhongshan Hospital, Fudan University, Shanghai, China.

**Keywords:** PLD2, invasion, metastasis, EMT, colorectal cancer

## Abstract

**Background and Objectives:** PLD2 has been identified as playing a critical role in cancer cell motility and migration and other pathophysiological processes. We investigated the expression of PLD2 and its biological functions and clinical implications in human colorectal cancer.

**Materials and Methods:** In this study, the expressions of PLD2 were analyzed in CRC cell lines and CRC samples by RT-PCR, western blot and immunohistochemistry. The PLD enzyme activity was studied using an PLD detection kit. We also performed matrigel invasion assay to evaluate the invasive capabilities in CRC cells. The expressions of EMT-related markers were quantified at mRNA and protein level using RT-PCR and western blot. We performed high-throughput RNA sequencing on PLD2 knockdown and overexpression CRC cell lines to explore the changes in gene expression associated with PLD2.

**Result:** Herein, we showed that PLD2 expression was relatively low in CRC cell lines and CRC samples and PLD2 deficiency was significantly correlated with more advanced clinical phenotype regarding lymphatic and distant metastasis and poor patient survival. We also detected that PLD2 knockdown favored epithelial-mesenchymal transition (EMT) and thus promoted CRC invasion and metastasis. Further exploration uncovered that the expressions of several important genes closely related to metabolic pathways in CRC were noticeably altered due to PLD2 deficiency, including ID1, IFIT4, OASL, IFIT2 and CTAG2.

**Conclusion:** Our results revealed that PLD2 deficiency promotes cell invasion and metastasis in CRC via EMT indicating PLD2 might have an important implication in carcinogenesis and progression and would be a new therapeutic target for cancer treatment.

## Introduction

Colorectal cancer (CRC) remains one of the most common human malignancies in the world, occurring in a significant percentage of the population. The prevalence and mortality of CRC continued to rise in the Asia-Pacific region and it has become the sixth primary cause of death induced by cancer in China because of living standard improvement and lifestyle change 1. Despite recent progress in the treatment strategies of CRC, the overall survival rate of colorectal cancer patients remains poor, mostly due to recurrence and metastasis 2. It is therefore an urgent need to further study the mechanism of colorectal cancer occurrence and progression to change this situation.

Epithelial-mesenchymal transition (EMT) is a reversible phenotypic change that occurs in solid tumors. Accumulating evidence suggests that the acquisition of EMT plays a key role in metastasis and is involved in carcinogenesis, invasion, and tumor recurrence, due to the loss of cell intercellular adhesion and epithelial polarization 3. EMT is controlled by a complex network of factors that includes signaling pathways, well-established transcription factors and cytokines, and the tumor microenvironment 4. The specific molecular mechanism of EMT in certain solid tumors has been a focus of study in recent years, but the conclusive evidence is still lacking.

Phospholipase D (PLD), which has been reported as relevant to various cancer cells, is an enzyme that catalyzes the hydrolysis of phosphatidylcholine (PC) to generate phosphatidic acid, a lipid second messenger that plays an important role in the signal transduction pathway in many physiological responses 5. PLD is becoming recognized as a critical regulator of survival signaling, cell proliferation, cell transformation, and tumor progression 6. In most mammalian cells, PLD exists as two isoforms, PLD1 and PLD2, which has attracted widely interest in recent years. However, up to now, there is no comprehensive report about the molecular mechanism underlying the regulation of PLD expression in colorectal cancer. Recent Studies have shown that high expression of PLD1 in colorectal cancer patients promotes Wnt signaling by selectively downregulating ICAT via the PI3K/AktTopBP1-E2F1 signaling axis 7. Although increased expression of PLD2 relates to proliferation, adhesion, invasion, and metastasis and have been observed in many malignant tumors, the role of PLD2 in human colorectal cancer still remains to be fully elucidated 8. Sondra and his colleagues have previously shown that PLD2 expression was increased in CRC and was correlated with lower survival rate and faster tumor growth. They also showed that PLD2 was secreted by cancer cells, altered the microenvironment and increased the secretion of specific SASP factors, which led to the promotion of stem cell features and the activation of Wnt signaling pathway 9.

As little information exists on how PLD2 function precisely effects tumor initiation and progression in CRC, it is of great importance to reveal a novel role of PLD2 in transformation and progression in colorectal cancer and provide the potential mechanisms. We therefore sought to evaluate whether PLD2 could be deduced as a potential detective biomarker in CRC samples and provided a new clue for seeking efficient treatment targets for metabolically abnormal late-stage and metastatic CRC patients.

## Materials and methods

### Participants and specimens

Fresh tumor tissue specimens and adjacent non-cancerous tissue specimens were obtained from colorectal cancer patients who underwent bowel resection at Zhongshan hospital of Fudan university. Tumor specimens were collected with informed consent obtained from all patients and procedures approved by institutional Review Board. Clinical stage was classified according to the tumor-node-metastasis classification of the International Union against Cancer. Histopathologic analysis was performed on hematoxylin and eosin-stained sections by routine pathologic examination to identify intravascular cancer embolus or perineuronal invasion, by experienced pathologists.

### Cell culture

The human colorectal cancer cell line (HT-29, SW480, SW620 and SW1116) and a normal colorectal epithelial cell line (NCM460) were purchased from Cell Bank of Chinese Academy of Sciences (Shanghai, China). We cultured HT-29 and NCM460 cells in a humidified incubator at 37°C and 5% CO2 with Dulbecco's modified Eagle medium (DMEM) supplemented with 10% fetal bovine serum (FBS) and penicillin (100U/ml)/streptomycin (100μg/ml). We cultured SW480, SW620, SW1116 cells in a humidified incubator at 37°C and 0.038% CO2 with L-15 medium (DMEM) supplemented with 10% fetal bovine serum (FBS) and penicillin (100 U/ml)/streptomycin (100μg/ml).

### Immunohistochemical staining (IHC)

Immunohistochemistry (IHC) for PLD2 was performed on 6mm-Formalin-fixed paraffin-embedded (FFPE) sections. PLD2 primary antibody (Proteintech, 1:50 dilution) was incubated overnight at 4°C. Immunostaining was visualized using diaminobenzidine and examined on a Zeiss fluorescence microscope and counterstaining was performed with hematoxylin. All IHC staining was assessed by two independent pathologists blinded to the patient's clinical and pathological data.

### PLD2 overexpression and shRNA transfection

PLD2 shRNAs were obtained from Institute of Biochemistry and Cell Biology, SIBS, CAS. A control scrambled shRNA was also purchased. Cell transfection was performed using polyethylenimine (PEI) transfection reagent (Sigma-Aldrich, St. Louis, MO, USA) according to protocols provided by manufacturers.

### Real time quantitative RT-PCR

Total RNA was extracted from cultured cells by using the Rneasy MiNi kit (Qiagen GmbH, Hilden, Germany), and then dissolved in diethylpyrocarbonate-treated (DEPC) water according to the manufacturer's instructions. cDNA was then synthesized using the Takara Reverse Transcription System Kit (Takara Biotechnology Co. Ltd., Japan), as described by the protocol. Real time quantitative RT-PCR were performed by using the Sybr green premix kit (BioRad, Hercules, California, USA). The GAPDH gene was used as a control housekeeping gene. All reactions were reproduced in triplicate.

### Western blot

Equal amounts of protein were separated by 10% sodium dodecyl sulfate-polyacrylamide gel electrophoresis (SDS-PAGE) and transferred to polyvinylidene fluoride (PVDF) membranes. Membranes were blocked in TBST containing 5% non-fat dry milk at room temperature for 1h, and incubated with PLD2, GAPDH primary antibodies at 4°C for overnight followed by incubation with horse-radish peroxidase (HRP) conjugated secondary antibodies. Immunoreactive bands were visualized using an enhanced chemiluminescent HRP substrate (Millipore, Billerica, MA, USA).

### Cell migration and invasion assay

CRC cell invasion assays were carried out in a transwell chamber precoated with 50μL of matrigel (BD Bioscience, Franklin Lakes, New Jersey, USA). Approximately 1×104 cells were resuspended in 100μl of FBS-free medium for 24h and plated into the upper chamber, while culture medium containing 10% FBS was added into the lower chamber. Non-invasive cells were removed from the upper surface of the membrane with a cotton swab, and the migrated cells that had adhered to the lower surface of the membrane were fixed with methanol and stained with crystal violet for 20 min. Cell numbers for invasion were then determined by counting the number of stained cells on the membrane under a light microscope (×200 magnification). The crystal violet staining was dissolved in 33% acetic acid and absorbance was measured at 570nm for quantification. The entire assay was repeated three times. The migration ability of CRC cells was evaluated by the wound healing assay. Cells were cultured in the 6-well dishes and allowed to grow to 80% confluence. Wounds were scratched using a sterile 200 µl pipette tip. After rinsed with PBS, cells were starved in serum-free medium. Cells were monitored every 6 hours. Cell photos were taken with a phase contrast microscope (×200 magnification).

### Cell proliferation assay

MTT assay was performed to detect cell proliferation according to manufacturer's instruction. CRC cells with PLD2 overexpression or knockdown were seeded into 96-well plates at a density of 104 cells/well. EGFP plasmid and shRNA were used as control. MTT solution (Sigma, Missouri, USA) of 10μl was added to each well at 24h intervals, followed by incubation at 37°C for 2h. After that, the MTT solution was removed carefully and 190μl DMSO was added to each well and plates were shaken for 10 minutes. At last, the cell proliferation was measured by reading absorbance at a wavelength of 450 nm with a microplate reader. The assays were repeated at least three times.

### Annexin V/PI assay

1×105 cells were harvested and resuspended in 200μl of 1X binding buffer with 5μl Annexin V at room temperature in the dark for 15 min. After that, cells were resuspended in binding buffer containing 5μl propidium iodide (PI), and immediately analyzed by flow cytometry on the 488nm laser to measure apoptosis and necrosis. This assay was repeated three times.

### RNA-Seq analysis

Total mRNA was isolated from each CRC cell line using Rneasy MiNi kit (Qiagen GmbH, Hilden, Germany) according to the manufacturer's instructions. RNA-Seq libraries were prepared with the TruSeq protocol (Illumina) and sequenced on the Illumina HiSeq2500 instrument using a 2×150 bp paired end read configuration. We aligned all the reads to the reference human genome (hg19) and determined expression levels, respectively. Differentially expressed genes had fold-change≥1.5 and adjusted P<0.05. Ingenuity Pathways Analysis (IPA) was used to identify biological pathways and functions associated with genes differentially expressed.

### Statistical analysis

Statistical analyses were performed using SPSS 22.0 (IBM, Chicago, IL). Relations between clinical parameters and PLD2 expression levels were analyzed using chi-squared test. *In vitro* studies were evaluated with student's t test. A P value less than 0.05 was considered statistically significant.

## Results

### Expression of PLD2 was relatively low in CRC cell lines and CRC samples

**Figure 1:**
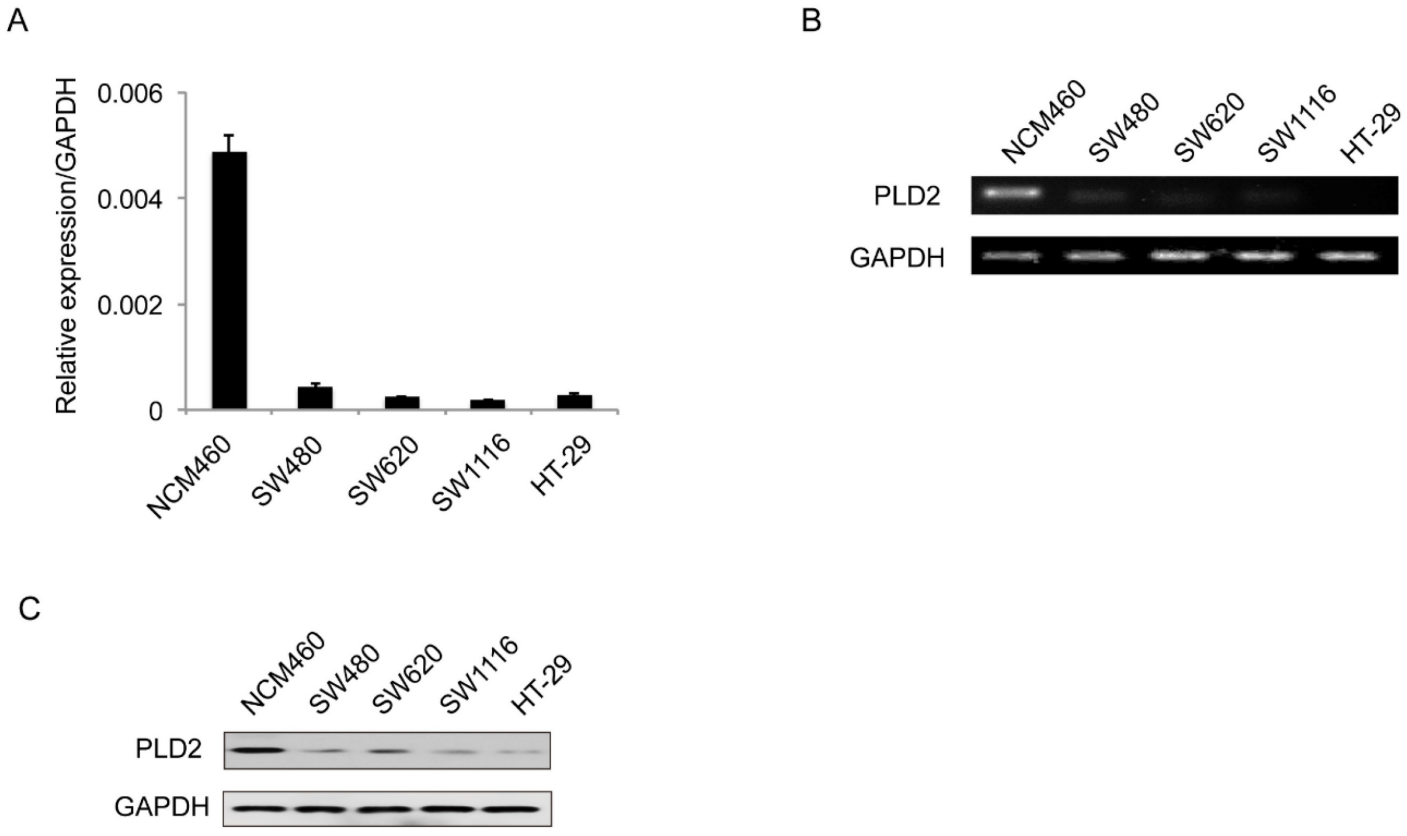
**Low PLD2 expression in CRC cell lines. (A)** Quantitative RT-PCR analysis of PLD2 expression in four CRC cell lines and non-CRC NCM460 cell line. **(B)** Semi-quantitative RT-PCR analysis of PLD2 expression in four CRC cell lines and non-CRC NCM460 cell line. **(C)** Western blotting analysis of PLD2 protein expression in four CRC cell lines and non-CRC NCM460 cell line.

**Figure 2:**
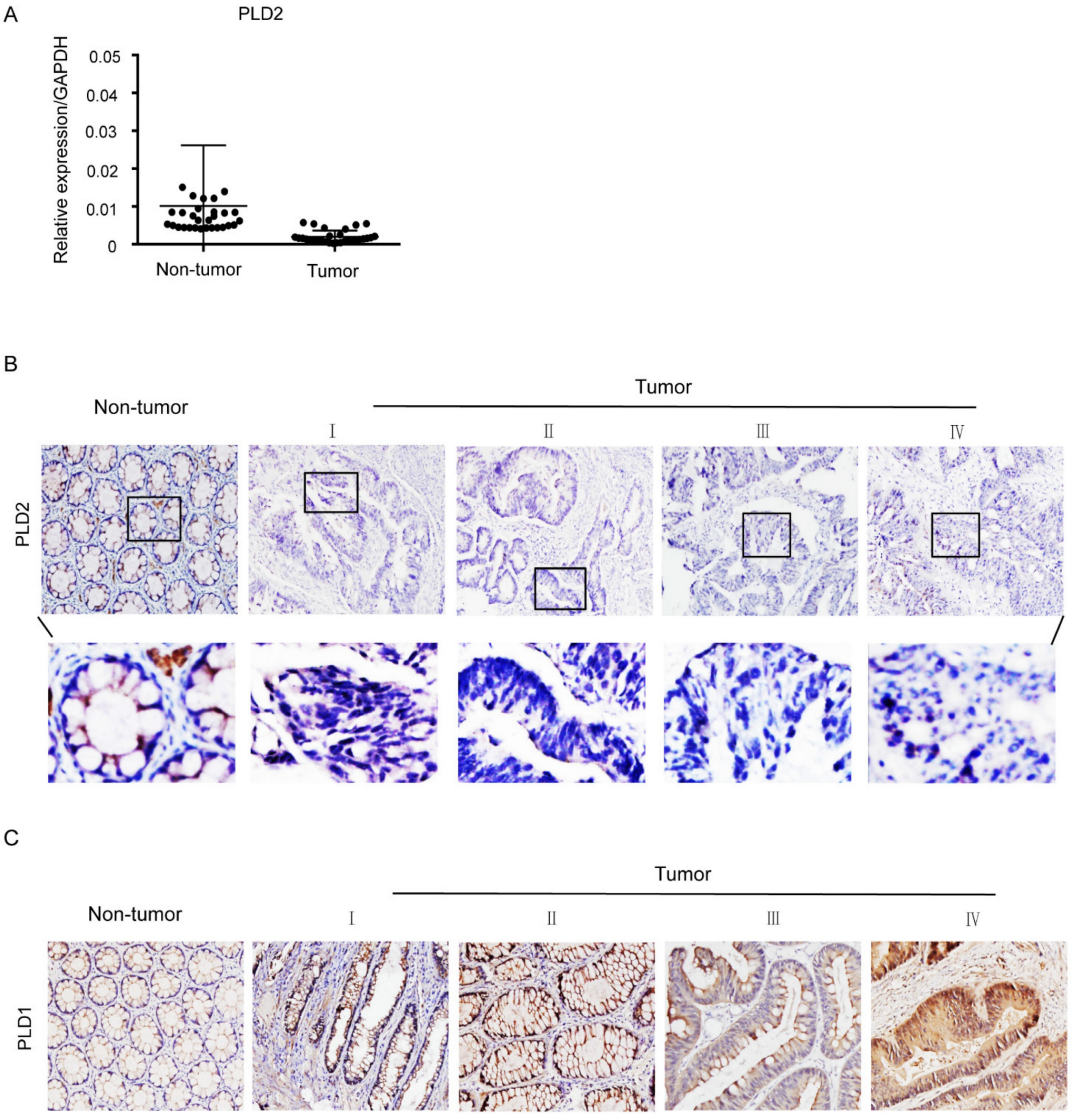
**Low PLD2 expression, high PLD1 expression in CRC tissues. (A)** Quantitative RT-PCR analysis of PLD2 expression in 67 pairs of colorectal tumors and its corresponding normal tissues. **(B)** Representative immunohistochemistry images showed varied but unanimously low expression of PLD2 in 67 pair of CRC samples. **(C)** Representative immunohistochemistry images showed varied but unanimously high expression of PLD1 in 67 pair of CRC samples.

PLD2 protein level in four CRC cell lines and normal colorectal mucosal epithelial cell line (NCM460) were examined by RT-PCR and western blot. In comparison to NCM460 cell line, a relatively low level of PLD2 was detected in all four CRC cell lines (SW480, SW620, SW1116, and HT-29) (Figure 1). For further verification, the expression levels of PLD2 were measured by quantitative RT-PCR in 67 pairs of CRC and adjacent noncancerous tissues. As shown in Figure 2A, CRC tissues showed a remarkably reduced level of PLD2 mRNA compared to adjacent normal tissues. Further immunohistochemistry assay displayed similar results of PLD2 and the varied overexpression of PLD1 in 67 CRC samples (Figure 2B and 2C).

### PLD2 expression was independent of PLD1 expression in CRC cells

**Figure 3:**
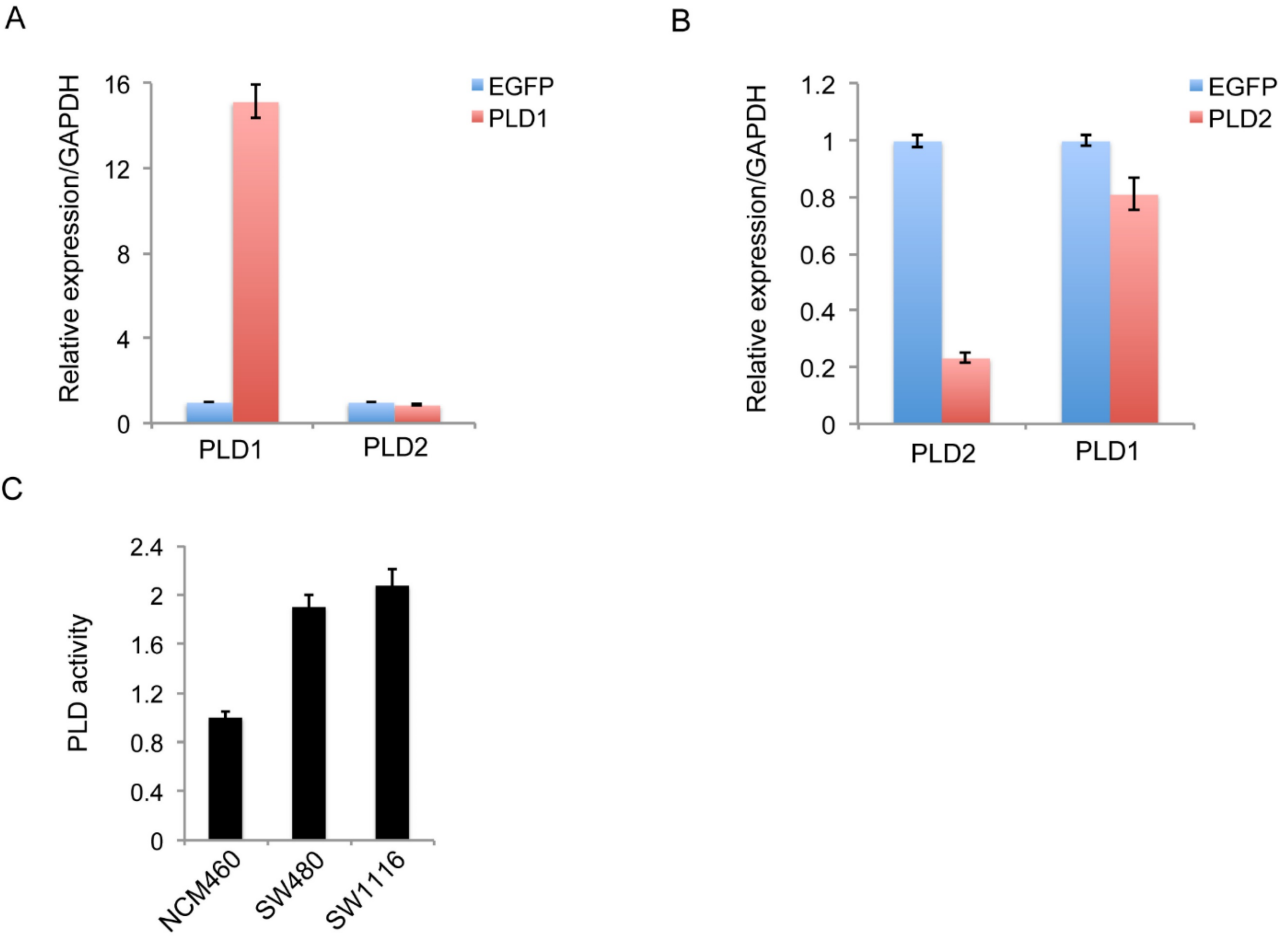
** The regulation of expression between PLD1 and PLD2 and high levels of PLD enzymatic activity in two CRC cell lines. (A)** Quantitative RT-PCR analysis of PLD2 expression in PLD1 overexpression stable cell line. **(B)** Quantitative RT-PCR analysis of PLD1 expression in PLD2 knockdown cell line. **(C)** PLD2 activity was significantly greater in two CRC cell lines than in non-CRC NCM460 cell line. However, there is only a small difference in PLD2 activity between in PLD1 overexpression cell line SW1116 and PLD2 knockdown cell line SW480.

To exclude the potential impact of significantly high PLD1 expression on the low level of PLD2, we constructed PLD1 overexpression stable cell line SW1116 and PLD2 knockdown cell line SW480. Further experimental results showed that the significantly high PLD1 expression weakly inhibit the expression of PLD2, which could not be comparable to the degree of inhibition we detected in clinical specimens (Figure 3A). Furthermore, the knockdown of PLD2 slightly inhibit PLD1 expression (Figure 3B). The PLD enzyme activity was studied using an PLD detection kit. PLD activity was shown significantly greater in two CRC cell lines compared with non-CRC NCM460 cell line (Figure 3C). However, there is only a small difference in PLD2 activity between PLD1 overexpression cell line SW1116 and PLD2 knockdown cell line SW480. Therefore, low-level expression of PLD2 in CRC patients was independent of high-level expression of PLD1.

### PLD2 knockdown promotes invasion and migration capacity in CRC cells

**Figure 4:**
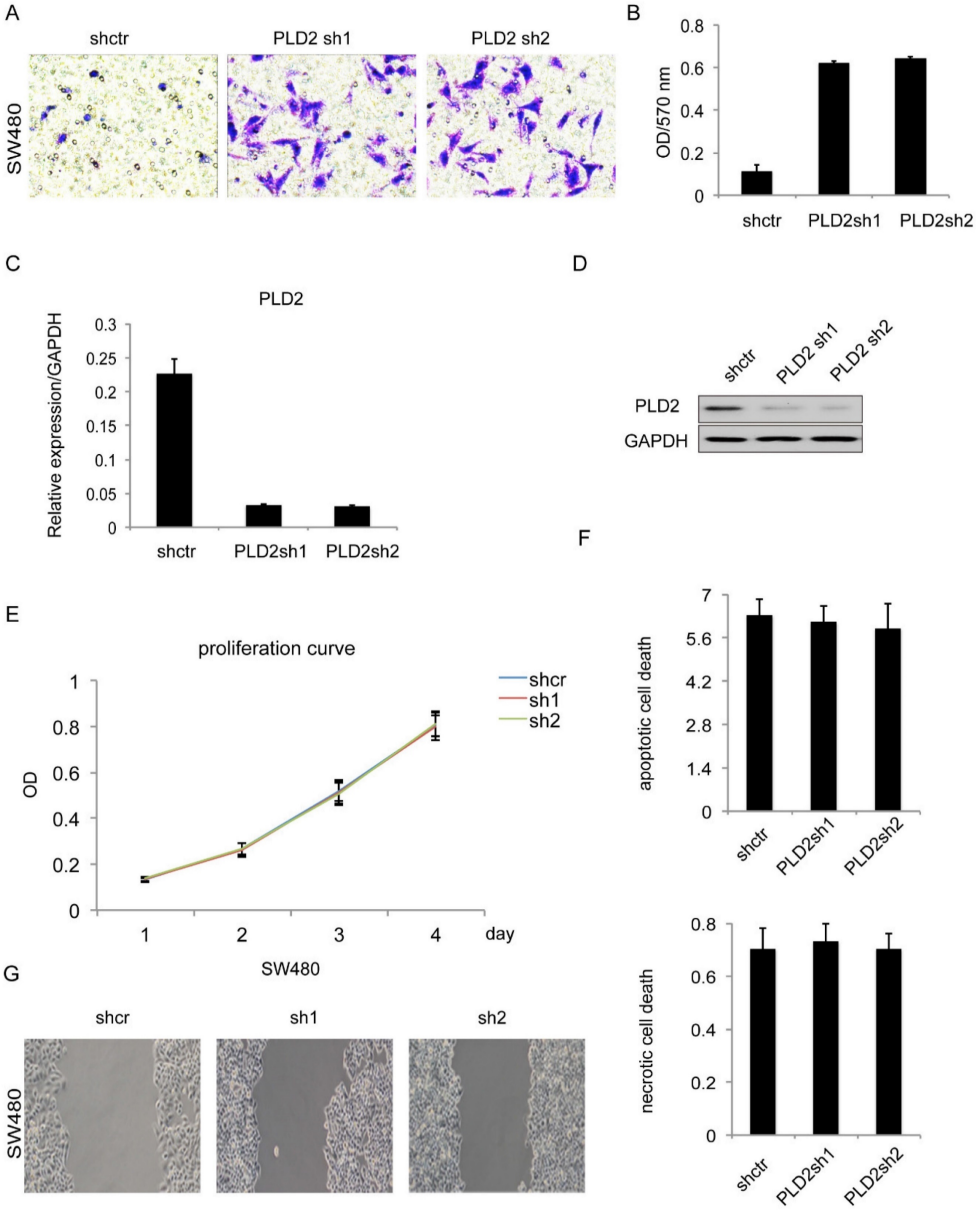
** PLD2 knockdown induced invasion and migration in CRC cell line SW480. (A)** Matrigel invasion assay showed that PLD2 knockdown strengthened invasive capacity. **(B)** Absorbance at 570 nm showed quantitative matrigel invasion data after 24 hours. (P<0.05, Student's t test). **(C)** Quantitative RT-PCR analysis of knockdown efficiency in PLD2 knockdown stable cell lines. **(D)** Western blotting analysis of knockdown efficiency in PLD2 knockdown stable cell lines. **(E)** MTT assay showed no obvious differences in proliferation when PLD2 is knocked down (Student's t test). **(F)** Annexin V/PI assay showed no obvious differences in apoptosis and necrosis when PLD2 is knocked down (Student's t test). **(G)** Wound-healing assay showed that PLD2 knockdown increased motility of CRC cells. The graph below showed mean opening distances after 24 hours (P<0.05, Student's t test).

In order to explore the biological functions of PLD2 in human CRC cells, a further experiment was performed by constructing PLD2 knockdown stable cell line through lentiviral infection. PLD2 knockdown efficiency was measured on both mRNA and protein level (Figure 4C and 4D). We performed both loss- and gain-of-function studies involving invasion, proliferation, apoptosis and necrosis. The results of matrigel invasion and Wound healing assay showed that decreased expression of PLD2 enhanced both invasion and migration abilities in SW480 cells (Figure 4A, 4B and 4G) whereas MTT assay and Annexin V/PI assay results demonstrated that it had no visible effect on proliferation, apoptosis and necrosis (Figure 4E and ​4F). Together, these results indicated that lower PLD2 expression enhanced tumor invasion *in vitro*, which played a pivotal role during CRC progression.

### PLD2 overexpression suppressed invasion and migration capacity in CRC cells

**Figure 5:**
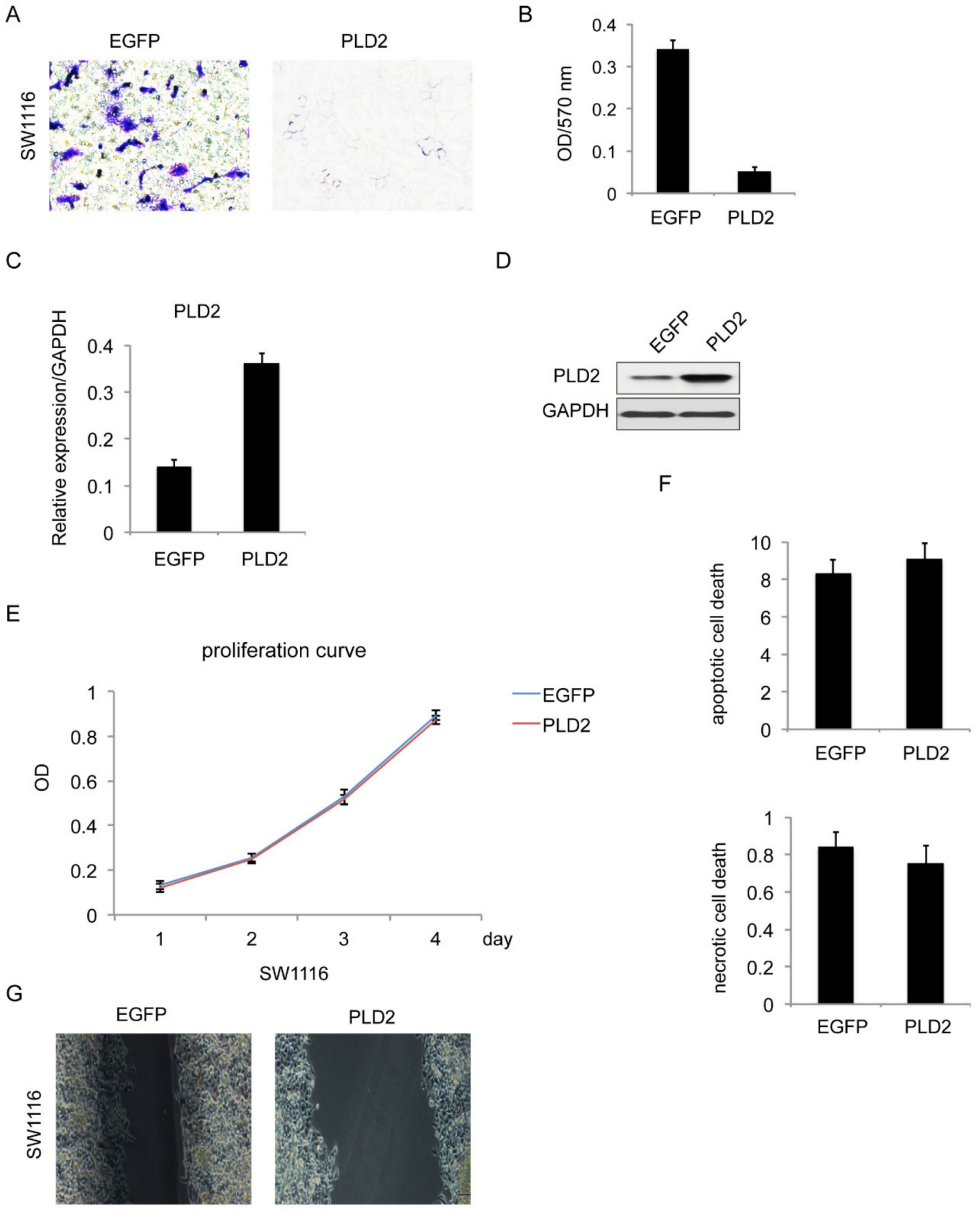
**PLD2 overexpression inhibited invasion and migration in CRC cell line SW1116. (A)** Matrigel invasion assay showed that PLD2 overexpression decreased invasive capacity. **(B)** Absorbance at 570 nm showed quantitative matrigel invasion data after 24 hours. (P<0.05, Student's t test). **(C)** Quantitative RT-PCR analysis of overexpression efficiency in PLD2 stable cell lines. **(D)** Western blotting analysis of overexpression efficiency in PLD2 stable cell lines. **(E)** MTT assay showed no obvious differences in proliferation when PLD2 is over expressed (Student's t test). **(F)** Annexin V/PI assay showed no obvious differences in apoptosis and necrosis when PLD2 is over expressed (Student's t test). **(G)** Wound-healing assay showed that PLD2 overexpression decreased motility of CRC cells. The graph below showed mean opening distances after 24 hours (P<0.05, Student's t test).

For further confirmation, similar experiments were performed with PLD2 overexpression stable cell line in SW1116 (Figure 5C and 5D). The result is consistent with prior data that PLD2 overexpression attenuated cell invasion and migration (Figure 5A, 5B​ and 5G) while having minimal influence on proliferation and cell death (Figure 5E and 5F​).

### PLD2 Knockdown favored Epithelial-Mesenchymal Transition (EMT) in CRC Cells

**Figure 6:**
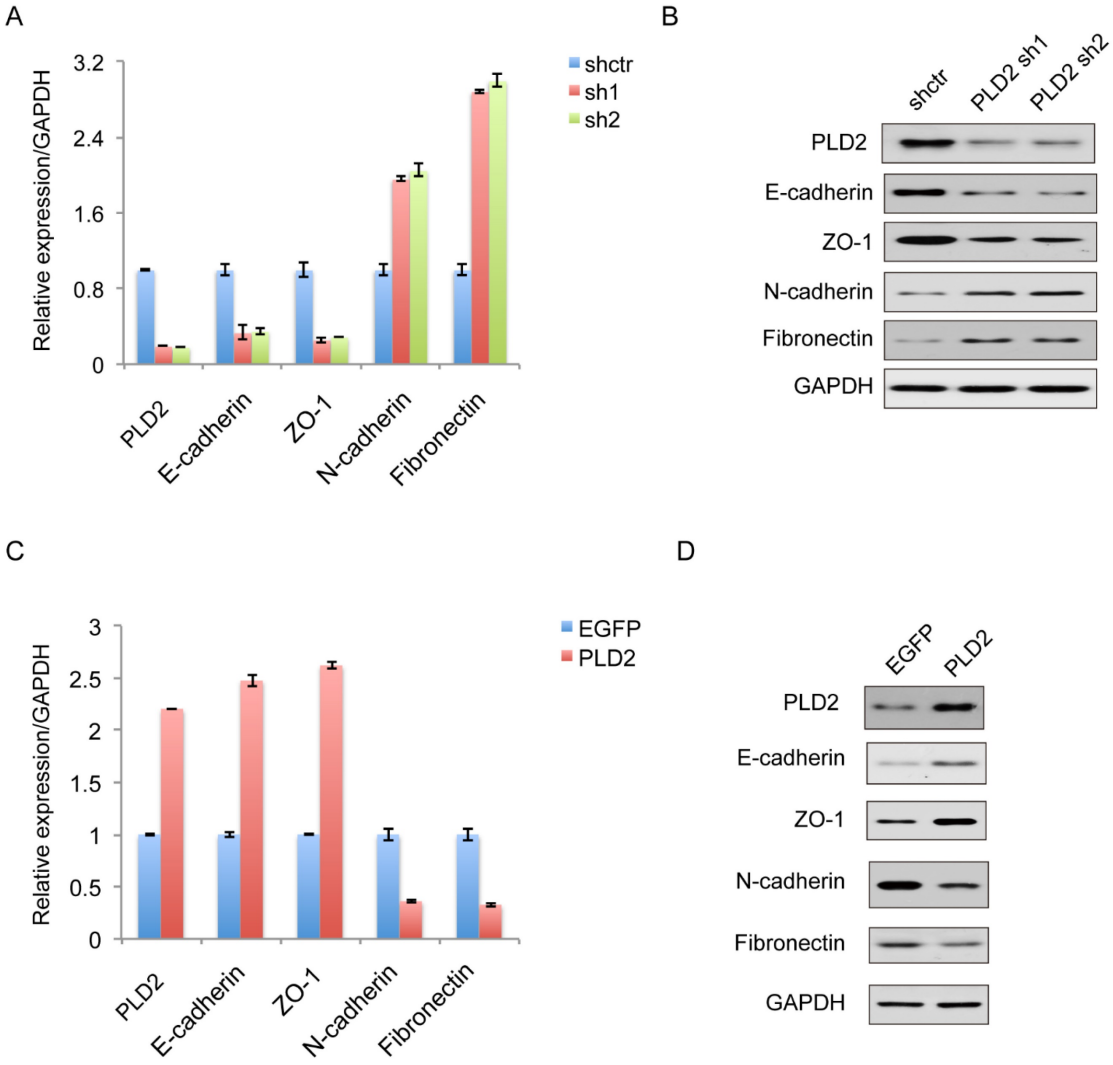
**PLD2 expression level regulated epithelial-mesenchymal transition (EMT) in CRC cell line. (A)** Quantitative RT-PCR analysis showed that PLD2 knockdown advanced EMT, as in down-regulation of epithelial markers and simultaneously up-regulation of mesenchymal markers. **(B)** Western blotting analysis showed that PLD2 knockdown advanced EMT. **(C)** Quantitative RT-PCR analysis showed that PLD2 overexpression suppressed EMT, as in up-regulation of epithelial markers and simultaneously down-regulation of mesenchymal markers. **(D)** Western blotting analysis showed that PLD2 overexpression suppressed EMT.

EMT plays a decisive role in the invasive and metastatic potential of many types of cancers. Therefore, further study is necessary to determine the relationship between PLD2 and EMT in CRC cells. On the molecular level, EMT is defined by the loss of epithelial marker E-cadherin and the acquisition of mesenchymal marker N-cadherin. As expected, decreased expression of PLD2 led to enhanced EMT, as evidenced by elevated expression of the mesenchymal markers N-cadherin (CDH2) and Fibronectin, and decreased expression of the epithelial marker E-cadherin (CDH1) and ZO1 (Figure 6A and 6B). Coherently, PLD2 overexpression had opposing effects (Figure 6C and 6D).

### PLD2 expression was correlated with prognosis and survival in CRC

**Figure 7:**
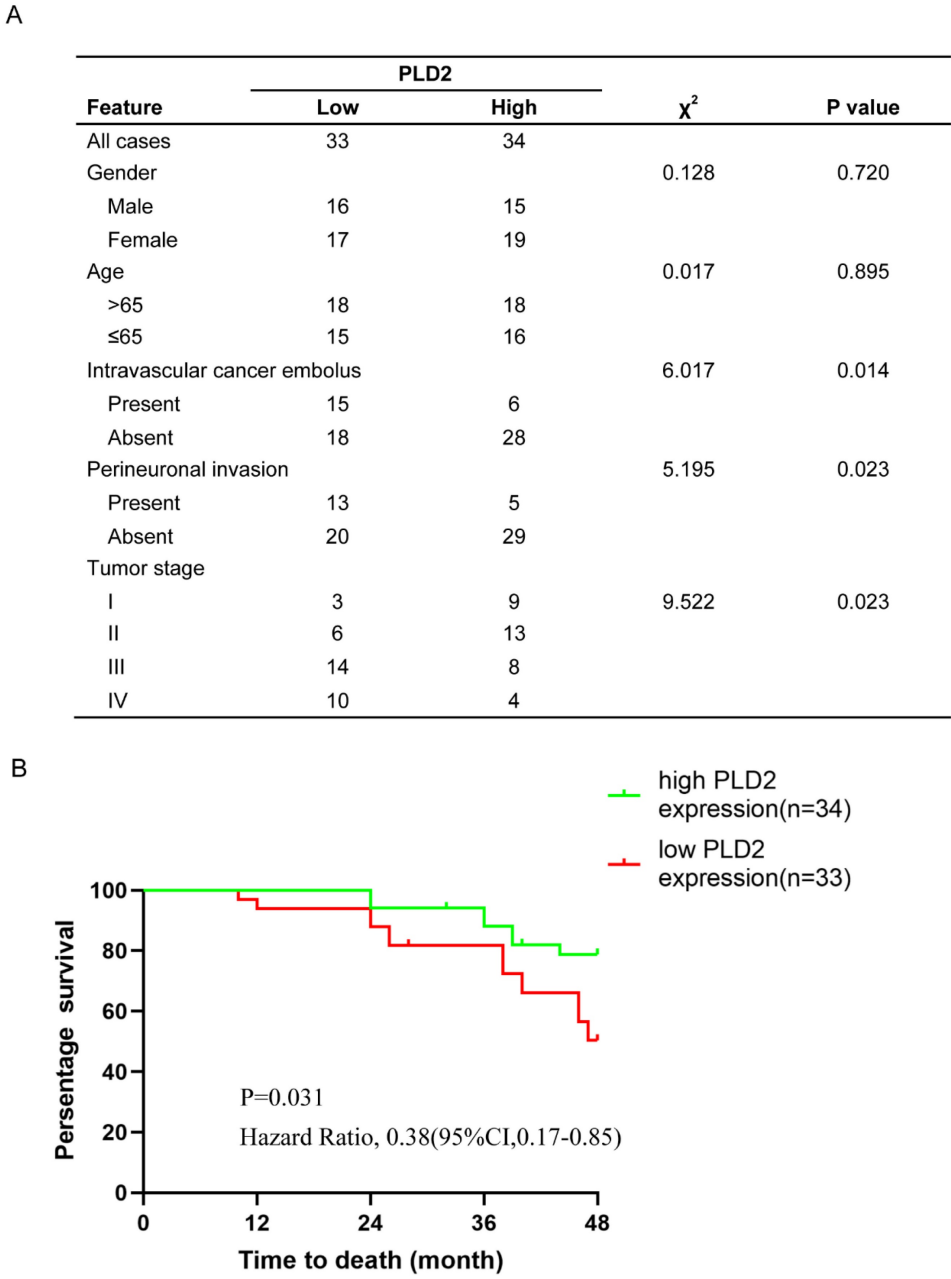
**The clinicopathological and prognostic significance of PLD2 expression level in CRC. (A)** Relationship between clinicopathologic findings and PLD2 expression in colorectal carcinoma. (The median expression level was used as the cutoff. Low expression of PLD2 in 33 patients was classified as values below the 50th percentile. High PLD2 expression in 34 patients was classified as values at or above the 50th percentile. For analysis of correlation between PLD2 and clinical features, Pearson's chi-square tests were used. Results were considered statistically significant at P<0.05.). **(B)** Kaplan-Meier survival analysis according to PLD2 expression in 67 CRC patients (P<0.05, log-rank test).

Our previous studies have verified low expression level of PLD2 in CRC samples compared to adjacent nontumorous tissues, yet its relevance with clinicopathologic features and prognosis remained to be evaluated. We performed tissue microarray on 67 pairs of CRC specimens and corresponding normal colorectal mucosal tissues. Further correlation analysis showed that the expression level of PLD2 was negatively correlated with CRC metastasis and advanced pathological TNM stage (Figure 7A). We also conducted survival analysis by using Kaplan-Meier curves and found that CRC patients with lower level of PLD2 survived for a significantly shorter time than patients with higher level of PLD2 (Figure 7B). The above evidence showed that PLD2 plays an important role in the process of CRC progression and could serve as a potential biomarker for metastatic CRC and an independent risk factor for poor prognosis.

### RNA sequencing in CRC cell lines

**Figure 8:**
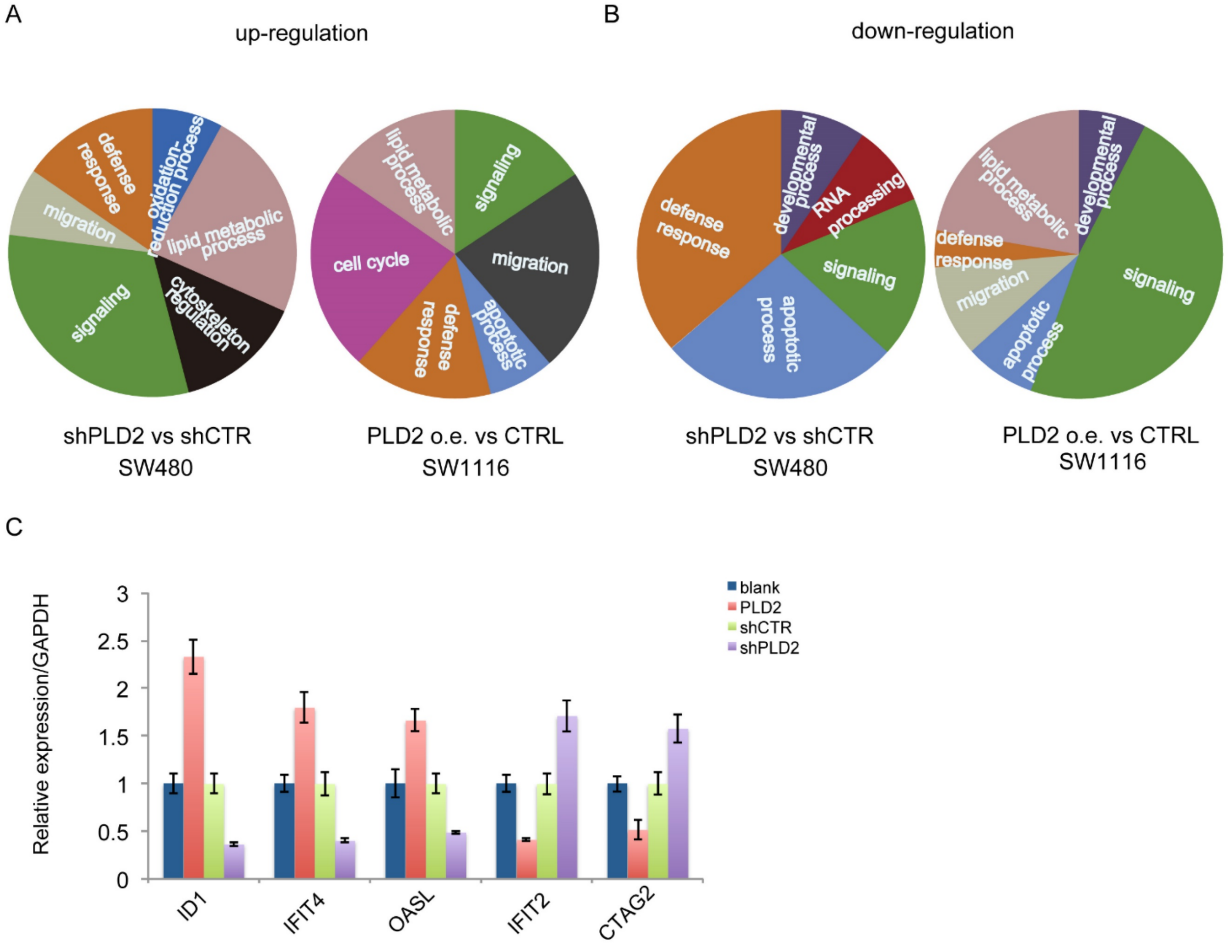
**PLD2 involved in the regulation of many cellular processes and led to a set of differentially expressed genes associated with CRC progression. (A)** The key functions associated with up-regulated genes in RNA sequencing clustering analysis on PLD2 knockdown CRC cell line SW480 and PLD2 overexpression CRC cell line SW1116. **(B)** The key functions associated with down-regulated expressed genes in RNA sequencing on PLD2 knockdown CRC cell line SW480 and PLD2 overexpression CRC cell line SW1116. **(C)** Quantitative RT-PCR analysis showed that PLD2 knockdown altered the expression of ID1, IFIT4, OASL, IFIT2 and CTAG2.

To get insights into the mechanism of function of PLD2 in CRC cells, high-throughput RNA sequencing (RNA-seq) was performed on PLD2 knockdown CRC cell line SW480 and PLD2 overexpression CRC cell line SW1116 to explore the gene expression profile associated with PLD2, rather than on the samples of CRC patients due to their high heterogeneities. The results of RNA-seq were consistent with the existing literature reports that PLD2 plays an important role in many cellular processes. Gene Ontology (GO) analysis demonstrated that the up-regulated gene were enriched related to important biological functions including signaling, lipid metabolic process, cytoskeleton regulation, defense response, migration, oxidation-reduction process, cell cycle and apoptotic process, whereas the down-regulated genes were enriched for defense response, apoptotic process, signaling, RNA processing, developmental process, lipid metabolic process and migration (Figure 8A and 8B). Moreover, several key genes strongly associated with reprogramming of cellular metabolism, invasion and metastatic spreading in human colorectal carcinoma were significantly differentially expressed in PLD2 knockdown and overexpression CRC cell lines compared to control cell lines. Then we chose some genes closely related to activation of important metabolic pathways in CRC, including ID1, IFIT4, OASL, IFIT2 and CTAG2, and confirmed the alteration of them by performing quantitative RT-PCR analysis. As expected, the results were basically consistent with RNA sequencing result (Figure 8C), which further confirmed the credibility of RNA sequencing results. Notably, the results showed that PLD2 knockdown significantly increased the expression of IFIT2 and CTAG2 and PLD2 overexpression increased the expression of ID1, IFIT4 and OASL, suggesting that these genes may be important target genes mediating PLD2 function. However, these results require further verification with additional cell experiments and animal experiments.

## Discussion

The 5-year survival rate for patients with early-stage CRC is about 90%, but this will decrease to 65% for tumors with regional spread and to 10% for patients with late-stage disease who have distant metastases 10. The detailed molecular mechanisms involved in the progression to CRC metastasis remain largely unknown, and therefore understanding the underlying mechanisms by which preinvasive CRC transits to invasive CRC is a top research priority and is of great significance to the development of more effective approaches to prevent tumor progression and ultimately improve patient outcomes.

An increasing amount of research shows that PLD activity may be particularly important in cell motility and migration, which play an indispensable role in the spread of cancer 11, 12. There are two classic mammalian isoforms of PLD: PLD1 and PLD2, which show quite different regulatory properties and subcellular localization. PLD2 displays a high basal activity and can be activated by a variety of upstream signals, whereas PLD1 has a low basal activity *in vitro* and can be regulated by small G proteins and PKC 13, 14. It has been shown that PLD2 is broadly localized to the plasma membrane, while PLD1 is usually located under steady-state conditions at the endosomes, Golgi complex, lysosomes, and secretory granules 15, 16. Recent evidence has continued to supported PLD as a critical component in many oncogenic signals, such as MAPK, RAS, and mTOR signaling 17, 18. Regarding CRC, PLD1 drives a positive feedback loop to reinforce the Wnt/beta-catenin/TCF signaling axis 19, however, the molecular mechanisms linking PLD2 and oncogenic signaling have not been addressed. A new study in Spain shows that PLD2, secreted by tumor cells induces senescence in neighbor fibroblasts, increases SASP factors expression and contributes to increased stemness of colorectal tumor cells by activating the Wnt pathway 9. But this study also mentions that the effect of PLD2 on tumor may be twofold: on the one hand, it can lead to tumor progress, but on the other hand, it can result in an arrest in tumor cell growth.

This study reveals, unexpectedly, that the expression of PLD2 in CRC cell lines and tissues was lower than that in the non-cancerous tissue, which was opposite to PLD1. This experiment has been repeated several times with similar results. The results of our study are quite different from those presented in existing literature 9, which represent that there may be significant differences in the expression and function of PLD2 in CRC cells among persons of different races, or it may be due to the differences in experimental systems and antibodies used. Moreover, this issue has been described in only a few publications and remains to be an area needing further research. Accordingly, it is of great importance to study how PLD2 functioned in tumor transformation and progression in colorectal cancer patients in China. Because regulatory relationship between PLD1 and PLD2 in CRC cells remains unclear, we could not exclude the possibility that high expression of PLD1 counteracts and reverses the suppressive role of low expression of PLD2 to sustain enzyme activity of PLD and thus PLD activity was increased as a whole in CRC patients. However, by constructing PLD1 overexpression cell line and PLD2 knockdown cell line, we found that PLD2 may possess unique regulatory functions that is independent of PLD enzyme activity in CRC cells, which suggested the need for further investigation. We speculated that PLD2 may play a “double-edged sword” role in the development of late-stage CRC carcinogenesis and metastasis, which is associated not only with the enzyme activity of PLD which drives progression of CRC, but also with other as-yet-undiscovered pathways which suppress the malignancy and the metastasis of tumor cells.

During tumor progression, epithelial cells within a local tumor lose cell polarity and cell-cell adhesion and acquire fibroblastoid properties that promote an invasive and metastatic phenotype by hijacking the developmental program of EMT, which occurs during embryogenesis, fibrosis and wound healing 20. This process is orchestrated by integrated networks of EMT-related genes, transcription factors (TFs) and different signal pathways, including TGFβ, Wnt, Gli, NFκB 21, 22. Similarly, in our subsequent studies, it has been shown that, PLD2 knockdown resulted in the boosting of EMT and the expressions of several key genes closely related to metabolic pathways important for tumor invasion and metastasis were noticeably altered under PLD2-deficient and PLD2-overexpressed circumstances in CRC cells. Results of this study showed that the expressions of ID1 23, IFIT4 24 and OASL 25 were decreased and the expressions of IFIT2 26 and CTAG2 27 were increased when PLD2 was knocked down. Previous studies indicated that ID1, as a SMAD-binding element (SBE) for TGFβ signaling target genes downstream targets, was transcriptionally repressed when TGFβ promotes the formation of an ATF3-Smad3/Smad4 complex 28. IFIT2, implicated as prognostic markers of clinical outcome for many cancers, can enhance the atypical PKC signaling pathway activation and subsequently promoted invasion of cancer cells through EMT 29. Taking into consideration these evidences, our future work will focus on identifying the exact mechanism by which PLD2 induced EMT and CRC progression. Consistent with these findings was the observation that PLD2 expression is negatively correlated with metastasis and prognosis in CRC.

## Conclusions

We provided the first direct evidence that PLD2 knockdown promotes invasion and metastatic capacity in CRC cells, at least in part by inducing EMT. Considering the fact that there are highly complex interactions among a variety of cancer-relevant pathways, PLD2 may act as a potentially promising prognostic marker or therapeutic target and be applied to prevention and treatment of advanced CRC. Nevertheless, a large number of studies should be performed in the future to explore the exact mechanism of the action of decreased PLD2 expression in advanced CRC and EMT-related pathogenesis.

## Abbreviations

CDH1: Cadherin 1
CDH2: Cadherin 2
CTAG2: Cancer/Testis Antigen genes 2
EMT: Epithelial-mesenchymal transition
ICAT: Inhibitor of β-catenin and T-cell factor
ID1: Inhibitor of differentiation/DNA binding 1
IFIT2: IFN-induced protein with tetratricopeptide repeats 2
IFIT4: IFN-induced protein with tetratricopeptide repeats 4
IHC: Immunohistochemistry
mTOR: Mammalian target of rapamycin
MAPK: Mitogen-activated protein kinase
OASL: Oligoadenylate synthetases-like
PC: Phosphatidylcholine
PI3K: Phosphoinositide 3-kinase
PLD: Phospholipase D
PKC: Protein kinase C
TCF: T-cell factor
TFs: Transcription factors
TGFβ: Transforming growth factor-β
ZO1: Zona occludens 1
